# Converting Three General-Cognitive Function Scales into Persian and Assessment of Their Validity and Reliability

**Published:** 2011

**Authors:** Payam Moin, Nima Khalighinejad, Arash Yusefi, Ziba Farajzadegan, Majid Barekatain

**Affiliations:** 1 General Practitioner, School of Medicine, Isfahan University of Medical sciences, Hezarjerib Street, Isfahan, Iran; 2 Associate Professor of Community Medicine, commmunity and preventive medicine department, school of medicine, Isfahan University of Medical sciences, Hezarjerib Street, Isfahan, Iran; 3 Associate Professor of NeuroPsychiatry, Department of Psychiatry, Noor University Hospital, Ostandari Street, Isfahan, Iran

**Keywords:** Disability Rating Scale, Glasgow Outcome Scale Extended, Galveston Amnesia and Orientation Test, Closed Head Injury, Cognitive function, General function, Rehabilitation

## Abstract

**Objectives:**

Glasgow Outcome Scale Extended (GOSE), Galveston Amnesia and orientation Test (GOAT) and Disability Rating Scale (DRS) are three popular outcome measure tools used principally in traumatic brain injury (TBI) patients. We conducted this study to provide a Farsi version of these outcome scales for use in Iran.

**Methods:**

Following a comprehensive literature review, Farsi transcripts were prepared by “forward-backward” translation and reviewed by subject experts. After a pretest on a few patients, the final versions were obtained. 38 patients with closed head injury were interviewed simultaneously by two interviewers. Main statistics used to assess validity and reliability included “Factor analysis” for construct validity, Cronbach’s alpha for internal consistency, and Pearson Correlation and Kappa Coefficient for inter-rater agreement.

**Results:**

Factor analysis for Farsi-GOAT (FGOAT) revealed 5 independent factors with a total distribution variance of 80.2%. For Farsi-DRS (FDRS), 3 independent factors were found with a 92.3% variance. The Cronbach’s alpha (95% confidence interval) was 0.84 (0.763- 0.919) and 0.91 (0.901-0.919) for FGOAT and FDRS, respectively. Pearson Correlation between total scores of two raters was 0.98 and 0.97 for FGOAT and FDRS, in order. Kappa coefficient (95% CI) between outcome rankings of raters was 0.73 (0.618-0.852) and 0.68 (0.594-0.770) for FGOAT and FDRS, respectively. As for Farsi-GOSE scale, Kappa value was 0.4 (0.285-0.507) for 8-level outcome ranking and improved to 0.7 (0.585-0.817) for 5-level scale. We found a good correlation between FDRS and FGOSE predicted prognoses (Spearman’s rho= 0.74, 95% CI: 0.676-0.802).

**Conclusions:**

FDRS and FGOAT had appropriate validity and reliability. The 8-level outcome FGOSE scale disclosed a low inter-rater agreement, but a suitable observer agreement was achieved when the 5-level outcome was applied.

## INTRODUCTION

Car accidents are the highest burden of disease in our country.^1^ Head trauma is the most common cause of morbidity and mortality among those affected by car accidents; as a result, treatment of head trauma plays an essential part in management of traffic accident victims. Following head trauma, victims suffer from cognitive, sensory and motor disorders that lead to a significant morbidity. Recognizing these injuries and determining the prognosis of victims during their treatment process can be very vital for the related specialists. Nowadays, several tools have been developed to evaluate the functional status of head trauma patients and their prognosis.^2^ Despite the high frequency of road accidents and head traumas in Iran, most of the existing tools have not been translated or standardized for use in Iran. In this study we translated and evaluated the validity and reliability of three cognitive and general function scales and adapt them for use in our population. These three tests were the following: Glasgow Outcome Scale Extended (GOSE), Galveston Amnesia and Orientation Test (GOAT), and Disability Rating Scale (DRS).

## METHODS

The objective of this study was to provide a Farsi version of three cognitive-general function scales and to evaluate them in terms of validity and reliability. These tests were GOSE, GOAT and DRS, which are being used to evaluate patients with head trauma.

DRS was originally developed in 1982 by Rapport et al.^3^ with the intention to monitor and evaluate patients’ general functional changes following severe head injuries, especially in an inpatient rehabilitation setting. This test consists of 8 main sections; the first questions, which are “eye opening”, “verbalization” and “motor response”, are a modified equivalent of the “Glasgow Coma Score (GCS)”. The next three items (“feeding”, “toileting”, and “grooming”) reflect the level of cognitive ability in self-care activities and the last two items (“level of function” and “employability”) address psychological adaptability and extent of handicap. As a result, questions of the DRS cover all three major parts of the rehabilitation and recovery definition of the World Health Organization, namely impairment, disability and handicap. The total score ranges from 0 to 29. The higher the score, the worse is the outcome of the patient. In addition to the primary survey of disabilities in head trauma patients, this test also tracks patient rehabilitation from coma to community.^4^

GOAT test has been designed and standardized by Levin et al. in 1979.^5^ This test is also devised to evaluate the cognitive function of patients with head trauma in their convalescence phase. It is especially useful in clinical settings to determine the duration of post-traumatic amnesia. This test comprises 16 questions. The score assigned to each question takes on a negative sign and is called error score. The total score equals 100 minus the sum of error scores. If the patient gets a total score of 75 or higher for two consecutive days, s/he is no longer supposed to be in the post-traumatic amnesia phase. If the score is between 66 and 75, s/he has a borderline status, and if the score is lower than 66, the patient would be still judged to be in the post-traumatic amnesia phase. This test is also considered as a complement to the Mini Mental State Exam (MMSE); MMSE is another test for evaluating cognitive function. In this instrument, the result is only valuable if the patient with head trauma has previously got a GOAT score of above 75.^6^

GOSE is a scale designed to evaluate the prognosis of patients with head trauma and assess their general functions, by placing them in specific categories. This test is being used by physicians in the convalescence phase of head trauma patients and is the extended form of the Glasgow Outcome Scale (GOS). GOSE was designed by Wilson et al. in 1998^7^ to address the limitations of GOS including broad categories with low sensitivity to change and lack of structured interview. GOS is a 5-level score, as follows; 1-Death, 2-Vegetative State, 3- Severely Disabled, 4-Moderately Disabled and 5- Good Recovery. In Extended GOS or GOSE, these were expanded to 8 modalities by dividing each of the three later outcome levels into upper and lower. This questionnaire has 8 groups of questions. The response to most of the questions is of the “Yes/No” type.^8^

We proceeded as follows: First, we contacted scale authors to acquire their permission and ask for their guidance through this project. Then, the latest version of the above mentioned tests were printed out from the available literature sources (Google scholar, Medline, Scopus and relevant textbooks). Every question in each instrument was annotated in the margins according to operations manuals and training materials, to establish a uniform performance among different interviewers.

We used the Forward-Backward translation method to translate our tests: at first, two physicians who were competent in English translated the tests into Farsi, after which two other competent physicians translated the tests back to English. Then, a common session was held in the presence of two experts of the English language and the original tests were compared with the translated ones to confirm the correctness, subject representativeness and fluency of the translations. Then as a pretest of the primary drafts, we asked medical interns to interview four patients each and fill out the forms. After that, the encountered problems were discussed in another meeting in the presence of interviewers (2 interns) along with a subject expert panel (2 psychiatrists, 2 neurologists and 2 neurosurgeons). Necessary modifications were made in the translated tools regarding the problems encountered in practice and the final Farsi versions of the tests were prepared.

After translating the tests, two interviewers were chosen among volunteer medical interns. Interviewers were required to attend two training sessions and successfully pass the final exam to ensure they have achieved the essential level of mastery to accomplish the tests independently.

Patients admitted for closed head injury in two university hospitals in Isfahan (Alzahra Hospital and Kashani Hospital) formed the target population. Samples were taken using convenience sampling method. Our inclusion criteria for the GOSE and DRS scales were as follows:

- Primary diagnosis of closed head injury.
- Age between 15 to 70 years.
- No other serious injury or previous disease, which could impress results on the opinion of examiner.

For GOAT the inclusion criteria were the same as above, with the addition of “GCS > 12” (as patients must be communicable enough to respond to questions). Both trained raters (interviewers) attended the interview session; however one of them asked the questions and the other one listened to the interview. At the end of the interview, the non-speaker rater could ask additional points to complete his own rating, if required. They interviewed in an alternate pattern. Interviewers stood apart and no conference was permitted among them. They filled in the questionnaires separately for each patient. The data from both interviewers were analyzed with SPSS 15, as explained below.

### Statistical analysis:

**DRS and GOAT**: For validity, “face and content validity” were examined in addition to a brief look at the “construct validity”. For face and content validity, we sent both translated and original scale formats to a panel of subject experts including 2 psychologists, 2 neurologists and 2 neurosurgeons to review and correct the Farsi format in terms of accurate translation, sensible appearance, representativeness and comprehensiveness of major concepts of interest.

“Principle component analysis”, a subset of the “Factor analysis” method, was employed to explore the latent structure of the variables in the scale related to “construct validity”. In this method, Varimax rotations were considered, Eigenvalue was set at one and the yielded factors from each questionnaire were clarified. To evaluate the “convergence construct validity”, the correlation between each question and the total score was measured using “item-total correlation” matrix; the “average item-total correlation” was reported. (Correlations above 0.6 were assumed appropriate)

As for reliability, we estimated “internal consistency” and “inter-rater agreement”. For “internal consistency” Cronbach’s alpha (within 95% confidence interval, more than 0.7 was considered acceptable) was determined. “Weighted Kappa” and “Pearson correlation” along with their 95% confidence interval, were measured to evaluate the “inter-rater agreement” for categorical outcomes and total scores respectively. As for Kappa coefficient, the amounts above 0.6 were considered appropriate. Kendall correlation coefficient was also calculated for FGOAT to compare our results to the original article.

**GOSE**: Because of the Yes/No type of questions and because of missing data, factor analysis was not possible for the FGOSE test. To confirm the reliability, Kappa coefficient was used to evaluate inter-rater agreement allowing outcome definitions for both FGOS and FGOSE. We then compared the ordinal outcomes of FGOSE and FDRS rated for each patient by the same interviewer. Spearman’s rho was calculated to accomplish this comparison.

In all the above statistics the p value was considered significant if it was <0.01.

## RESULTS

FDRS and FGOSE tests were completed in 38 patients (6 female and 32 male, mean age 34 years, mean injury-test interval 6.7 days, median of 5 years education, and mean GCS of 13.5 at the time of examination). The FGOAT test was performed on 34 patients (6 female and 28 male, mean age 35.6 years, mean injury-test interval 7.2 days, median of 5 years education, and mean GCS of 14 at the time of examination).

FDRS: Table 1 shows the factor analysis for FDRS reflecting hidden conceptual domains within the test structure. “Factor analysis” divided the questions into three categories, with 92.3% variance. The first factor included questions 4, 5, and 6, and concerned daily activities including feeding, toileting and grooming. The second factor consisted of three questions (Question 1, 2 and 3) similar to GCS scale (eye opening, verbal and motor response). The third factor was about the social and emotional function of the patients and was made up of two questions (Question 7 and 8). The average item-total correlation (Standard Deviation (SD)) was 0.72 (0.13) and 0.73 (0.13) for raters A and B in order, revealing a suitable consistency between the score of each question and the total score.

**Table 1:** Disability Rating Scale (DRS) factor analysis; First Factor: Daily activities (Feeding, Toileting, Grooming; Second Factor: Communication skills; Third Factor: Social and emotional activities

DRS factor analysis	First factor	Second factor	Third factor
Question 6	92%		
Question 5	92%		
Question 4	91%		
Question 3		96%	
Question 1	45%	79%	
Question 2	58%	63%	
Question 8			94%
Question 7			91%

For reliability, the Cronbach’s alpha (95% Confidence Interval (CI)) was 0.91 (0.901-0.919). Pearson correlation between the total scores reported by two raters was significant (0.97) and Kappa coefficient between the outcomes reported by two raters was 0.68 (95% CI: 0.594-0.770), verifying an acceptable level of agreement between raters.

FGOAT: Factor analysis was done for the FGOAT test and showed that five independent factors might be procured from this test, which consists of 80.2% of the total distribution variance. The first factor comprised 6 questions that were related to place orientation and patient’s memory of the events prior to the accident. The second factor, which included two questions, was associated to the events taking place after the accident. The third factor, with two questions, was related to personal information. The fourth factor asked about the present time in year and month, and consisted of two questions. The fifth factor, which was assessed by three questions, was also related to time but in terms of hours and questioned how the patient had been transferred to the hospital as well. Table 2 shows the set of questions assigned to each extracted factor of factor analysis. As can be seen, the questions 4, 5, 6, and 14, encompass more than one factor; especially question 4, in which the response affects three factors. The average (SD) of item-total correlations was 0.6 (0.12).

**Table 2:** Galveston Amnesia and Orientation Test (GOAT) factor analysis; First Factor: Place orientation and remembering events before trauma; Second Factor: Remembering events after trauma; Third Factor: Personal information; Fourth Factor: Time orientation (Year and month); Fifth Factor: Time orientation (Hour) and hospital transportation after trauma

GOAT factor analysis	First Factor	Second Factor	Third Factor	Fourth Factor	Fifth Factor
Question 11	90%				
Question 10	90%				
Question 14	67%				
Question 6	65%	41%			
Question 5	59%	40%			
Question 4	50%	4%		48%	
Question 8		91%			
Question 9		84%			
Question 3			93%		
Question 1			93%		
Question 2			53%		
Question 15				87%	
Question 16				87%	
Question 13 Question 12					81%
Question 7					79%
					67%

Statistical methods used to assess reliability of FGOAT were exactly the same as those of FDRS scale. The Cronbach’s alpha (95%CI) was 0.84 (0.763- 0.919). Pearson correlation between total scores rated by raters was 0.98. As for measure of agreement in outcome between raters, Kappa coefficient was 0.73 (95% CI: 0.618-0.852). Kendall correlation coefficient between prognoses defined by raters was 0.84 (95% CI: 0.774-0.914).

Worth mentioning here is that we appended a clause to the GOAT score interpretation material; as many patients with head trauma are from a low educational or illiterate population, they are rarely oriented in date. This made it impossible to understand whether their mistakes in date-related questions arose from head trauma or not. In these cases, we proposed a simple formula to judge the total score, which is attached to the end of our FGOAT scale.

FGOSE: Kappa coefficient was used to assess correlation of outcomes between raters; Kappa coefficient for the 8- level outcome scale was 0.4 (95% CI: 0.285-0.507) whilst for the 5-level GOS, it was 0.7 (95% CI: 0.585-0.817). Overall agreement on final outcome between raters was 66% for 8-level and 87% according to 5-level scale.

FDRS and FGOSE outcome comparison: To compare ordinal outcomes between DRS and GOSE we measured “Spearman’s rho”, which was 0.74 (95% CI: 0.676-0.802).

## DISCUSSION

Because of the high physical and cognitive damages following head injuries, prognostic tests are of great value in the management and rehabilitation of these patients. However, cognition is a complex and general term, which literally refers to processes of thought, encompassing various concepts including thinking, problem solving, perception, memory and reasoning. Thus, we tried to arrange a battery of cognitive scales that represent as many domains of cognition as possible. DRS, GOSE and GOAT do not purely evaluate cognitive performance; they rather give out a mixed general-cognitive estimation. This is completely justifiable since they are used to probe patients’ recovery after head trauma that involves both physical and cognitive rehabilitation.

FDRS: Factor analysis separated three factors for FDRS, containing 92.3% distribution variance, and showed desirable construct validity. Rapport et al (1982) obtained a similar Pearson correlation coefficient for inter-rater reliability of DRS as we did (0.97 vs. 0.96).^3^ Gouvier (1987) also reported the same correlation (0.98).^9^

However, they compared scores of three raters whereas we had two raters. We also obtained a 0.68 Kappa coefficient indicating a good inter-rater agreement in outcome. Although we employed the 0.5 rating DRS version, the Traumatic Brain Injury Model Systems National Database members voted to use prior integer-number format after 2010.

FGOSE: We obtained a 0.4 Kappa value for inter-rater reliability of FGOSE, which is much lower than the 0.85 in its original article.^7^ Nevertheless, based on the GOS outcome classification, we calculated a Kappa coefficient between raters of 0.7. These results are similar to the 0.48 and 0.77 Kappa values that Maas et al reported for GOSE and GOS respectively in “live” situation.^10^ Overall agreement in outcome between raters was 66% (based on GOSE outcome) and 87% (based on GOS outcome) in our study, which is parallel to the 78% and 92% of the original article.^7^ Smith (1979) obtained a 0.85 correlation between DRS and GOSE at discharge.^11^ Our results revealed a “Spearman’s rho” of 0.74, which is suitable as well.

FGOAT: Levin and colleagues^5^ found a 0.99 (p<0.001) Kendall (T) correlation as the degree of similarity between two sets of ranks given by two raters. We had a Kendall value of 0.84, which is close to their result and appropriate. According to factor analysis, construct validity in factor analysis separated five factors for FGOAT with 80.2% distribution variance, which shows a good underlying content structure for FGOAT.

The International Test Commission provides guidelines for psychiatric test translation and development since 1992.^12^ Although we tried to perform this study by conforming to these guidelines, there were some weak points in our study. The first limitation was the relatively small sample size. Furthermore, it would have been preferable to use a standard test to evaluate the validity of all these tests, which was however not possible due to lack of access to such a test. Another weak aspect of our study was the lack of repeatability assessment for reliability of the tests; this was in part because of the fast physical and mental improvement in severe head trauma patients in addition to oscillations in orientation level, so that we could not repeat the test after some days on the same person expecting the same results.

We attained compelling results regarding reliability and validity of FDRS and FGOAT, introducing them as a valuable instrument to predict the prognosis of traumatic brain injury in the Iranian population. FGOSE carried a 0.4 Kappa value for its 8-level outcome, which was not suitable. However, the Kappa value of 0.7 for the 5-level outcome system was more promising. Consequently, we suggest applying the primary 5-level outcome rating method to provide a better observer agreement in the FGOSE scale. Recently, a study targeted the problem of high variation among different raters of the conventional GOSE score interpretation system and proposed an alternate GOSE rating system, intended to minimize the inter-rater variations.^13^ Rigorous training in structured interviewing and monitoring the quality of assessment together with providing feedback to interviewers are undeniably important for more consistent outcomes.
